# Synthesis and Properties of pH-, Thermo-, and Salt-Sensitive Modified Poly(aspartic acid)/Poly(vinyl alcohol) IPN Hydrogel and Its Drug Controlled Release

**DOI:** 10.1155/2015/236745

**Published:** 2015-08-09

**Authors:** Jingqiong Lu, Yinhui Li, Deng Hu, Xiaoling Chen, Yongmei Liu, Liping Wang, Yansheng Zhao

**Affiliations:** ^1^School of Chemical and Biological Engineering, Taiyuan University of Science and Technology, Taiyuan 030024, China; ^2^College of Chemistry and Chemical Engineering, Taiyuan University of Technology, Taiyuan 030024, China; ^3^Sansom Institute for Health Research, School of Pharmacy and Medical Sciences, University of South Australia, Adelaide, SA 5001, Australia

## Abstract

Modified poly(aspartic acid)/poly(vinyl alcohol) interpenetrating polymer network (KPAsp/PVA IPN) hydrogel for drug controlled release was synthesized by a simple one-step method in aqueous system using poly(aspartic acid) grafting 3-aminopropyltriethoxysilane (KH-550) and poly(vinyl alcohol) (PVA) as materials. The hydrogel surface morphology and composition were characterized by Fourier transform infrared spectroscopy (FTIR) and scanning electron microscopy (SEM). The thermal stability was analyzed by thermogravimetric analysis (TGA). The swelling properties and pH, temperature, and salt sensitivities of KPAsp, KPAsp/PVA semi-interpenetrating polymer network (semi-IPN), and KPAsp/PVA IPN hydrogels were also investigated. All of the three hydrogels showed ampholytic pH-responsive properties, and swelling behavior was also extremely sensitive to the temperature, ionic strength, and cationic species. Finally, the drug controlled release properties of the three hydrogels were evaluated and results indicated that three hydrogels could control drug release by external surroundings stimuli. The drug controlled release properties of KPAsp/PVA IPN hydrogel are the most outstanding, and the correlative measured release profiles of salicylic acid at 37°C were 32.6 wt% at pH = 1.2 (simulated gastric fluid) and 62.5 wt% at pH = 7.4 (simulated intestinal fluid), respectively. These results indicated that KPAsp/PVA IPN hydrogels are a promising carrier system for controlled drug delivery.

## 1. Introduction

Hydrogels are natural or synthetic hydrophilic networks of polymer, which have strong ability to retain water and other biological fluids, while preserving their shapes [1, 2]. Over the last two decades, stimulus-responsive hydrogels have been extensively studied on their reversible volume changes controlled by external stimuli, such as pH, temperature, solvents, ionic strength, and ultrasonic sound [3, 4]. Their porosity and responsiveness to the environment are vital in the biological pharmaceutical applications, especially for drug delivery. The pH-responsive hydrogels are sensitive to the environment and have been broadly developed, due to their large variations in physiological pH at various body sites in normal as well as pathological conditions [5–8]. Because thermosensitive hydrogels easily dissolve in solutions at low temperature, they have attracted attention. Furthermore, they can be easily separated from solution if the lower critical solution temperature is lower than the environment temperature [8–10]. As a result, these hydrogels have been broadly applied to the development of novel drug carriers, which exhibited controlled-release characteristics [11–14].

Due to their protein-like structures, poly(aspartic acid) hydrogels have significant advantages over other polymers, such as biocompatibility, biodegradability, and toxicological suitability (lack of toxicity, antigenicity, and immunogenicity) [15]. On the other hand, poly(aspartic acid) hydrogel enjoys superior swelling property, pH sensitivities, and thermosensitivities due to the free carboxylic acid and amino groups existing in its structure. On account of these dramatic properties, poly(aspartic acid) has become an attractive candidate for drug carriers [16]. Nevertheless, poly(aspartic acid) hydrogel still has the weakness of poor salt tolerance and inferior swelling property in alcohol solution due to its single component and structure, which leads to the limited drug-loaded capacities of the hydrogel since most of drugs are alcohol-soluble.

Interpenetrating polymer network (IPN) is a mixture of two or more cross-linked networks, which are dispersed or mixed together at a molecular segmental level without covalently bonding [17]. The networks are entangled and concatenated together, which cannot be separated without breaking the chemical bonds. Therefore, the properties of each network may be well reserved and the proportion of each polymer can be adjusted individually. The IPN technique has been considered to be an effective method to obtain materials with better improved combination of the properties of their components [18], whilst preserving their remarkable biodegradation and biocompatibility.

As another important material, PVA is a polyhydroxy and water-soluble polymer. It was easily formed film by solution casting and has good hydrophilic properties and high elasticity [19, 20]. Due to these satisfactory physical characteristics and its good biocompatibility, PVA has been broadly applied in the biomedical and pharmaceutical areas and is widely explored for hydrogel fabrication [21, 22]. In particular, because of containing the large amount of hydroxyl, PVA can improve not only the salt tolerance of polymers, but also their swelling property in alcohol solution.

Based on poly(aspartic acid), IPN hydrogels have been developed in different areas, such as biotechnology and medical and pharmaceutical applications. Liu et al. prepared a pH-sensitive semi-IPN hydrogels by using konjac glucomannan (KGM) and poly(aspartic acid) with trisodium trimetaphosphate (STMP) as the cross-linking agent [16]. Yang et al. synthesized different IPN hydrogels by varying the compositions of xanthan gum (XG) and poly(aspartic acid) cross-linked with 1,6-Hexanediamine in dimethylformamide (DMF) [23]. To obtain pH-sensitive IPN hydrogels for drug controlled release, Liu et al. introduced PVA hydrogel into poly(aspartic acid) hydrogel by using a two-step method with six-time freeze-thawing treatment [24]. The swelling and drug release characteristics of the prepared pH-sensitive IPN hydrogel were weakened due to the compact structure of the IPN hydrogel network formed during the incorporation of PVA. Therefore, a simple one-step method was designed to prevent the IPN hydrogel network structure from compacting, in which the self-cross-linking reaction of KPSI and PVA occurs simultaneously.

The aim of this paper is to introduce a simple one-step method to synthesize modified poly(aspartic acid) and PVA IPN hydrogels in an aqueous system that satisfied drug controlled release. In this study, a kind of modified polymer polysuccinimide (PSI) grafted by KH-550 (KPSI) with silicon hydroxyls was prepared by grafted 3-aminopropyltriethoxysilane (KH-550) to PSI. KPAsp hydrogel was obtained through KPSI hydrolysis and self-cross-linking. The following novel KPAsp/PVA hydroxyls with semi-INP and IPN structures were prepared by a simple one-step method in an aqueous system. FTIR and SEM were used to characterize the hydrogel composition and surface morphology. Salt, temperature, and pH sensitivities were investigated through measuring equilibrium swelling ratios in different environmental solutions. Furthermore, the drug release properties of the three hydrogels were studied. As far as we know, little has been reported about this aspect of study.

## 2. Experimental

### 2.1. Materials

PSI was prepared in our laboratory [25] (molecular weight: 100,356 Da, polymerization degree: 872). DMF, PVA, and ethanol absolute were purchased from Tianjin Kemiou Chemical Regent Company, China. Sodium hydroxide (NaOH), 3-aminopropyltriethoxysilane (KH-550), sodium chloride (NaCl), and glutaraldehyde were purchased from Tianjin Fengfan Technology Chemical Regent Company Ltd., China. Ferric trichloride and anhydrous calcium chloride were obtained from Tianjin Damao Chemical Regent Company Ltd., China. All reagents were of analytical reagent grade.

### 2.2. Synthesis

#### 2.2.1. Synthesis Mechanism of KPAsp/PVA Hydrogel

**Figure 1:**
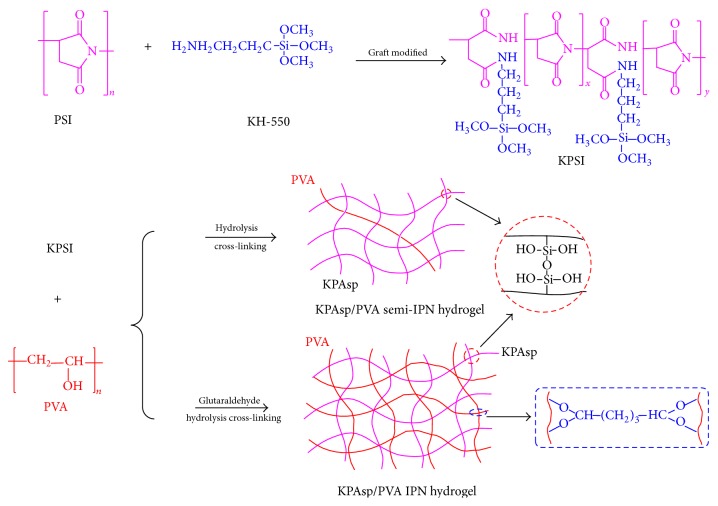
Schematic of KPAsp/PVA semi-IPN and IPN hydrogels preparation.

The synthesis mechanism of KPAsp/PVA semi-IPN and IPN hydrogels was illustrated in Figure 1.

#### 2.2.2. Synthesis of KPSI

1 g of PSI and 10 mL of DMF were simultaneously placed in a 100 mL beaker under magnetic stirring at 35°C. After PSI was completely dissolved, an amount of 1.8 mol% of KPSI was added into the solution and maintained with the same stirring speed and temperature for 3 h. Then, 20 mL of alcohol was added into the beaker with vigorous stirring. Subsequently the precipitate was separated by filtration and dried at 50°C in a vacuum oven. At last, KPSI was obtained.

#### 2.2.3. Synthesis of KPAsp Hydrogel

One gramme KPSI was dispersed into 20 mL deionized water and heated to 35°C. Then, NaOH (with a concentration of 2 mol/L) was added drop-wise into the solution to maintain the hydrolysis reaction at pH 10 for 4 h. The solution was put in a vacuum oven at 75°C for further reaction for another 2 h. And then, alcohol was poured into the solution, and a precipitate was obtained and collected by filtration and dried at 50°C in a vacuum oven. Finally, KPAsp hydrogel was obtained.

#### 2.2.4. Synthesis of KPAsp/PVA IPN Hydrogel

Similarly, 1 g of KPSI was dispersed into 20 mL deionized water. Then 24.5 mL aqueous solution of PVA (containing 0.5 g PVA) was poured into the KPSI solution with agitation and heated to 35°C. Next, NaOH (with a concentration of 2 mol/L) was added drop-wise into the solution to maintain the pH at 10 for 4 h. Half an hour before hydrolysis reaction completed, the cross-linker and glutaraldehyde (3.38 mol% based on PVA) were added. The solution was put in a vacuum oven at 75°C for further reaction for 2 h. Afterwards, alcohol was added to the solution and the precipitate was separated by filtration and dried at 50°C in a vacuum oven. Thereby the KPAsp/PVA IPN hydrogel was obtained. KPAsp/PVA semi-IPN hydrogel was also prepared in the same manner without the addition of glutaraldehyde.

### 2.3. Characterization

The composition of hydrogels was investigated by using FTIR. The vacuum-dried hydrogel samples were dispersed in dry KBr powders. The spectra were recorded from 4000 to 500 cm^−1^ with a resolution of 2 cm^−1^ by a FTIR spectrometer (Shimadzu-8400S FTIR, Japan).

ASEM (MIRA3, UK) was used to determine the surface morphology of dry hydrogels. The hydrogel sample was ground by a mortar and pestle, and the sample powder was sputter-coated by palladium/gold.

The thermal stability of each hydrogel was evaluated using a thermogravimetric instrument (TGA-50, Shimadzu, Japan). The samples were heated from 40 to 600°C under an N_2_ atmosphere flow at a rate of 100 mL/min at a heating rate of 10°C min^−1^.

### 2.4. Swelling Behavior Study

The measurement of the swelling ratio of the hydrogels was conducted at 30°C by a tea-bag (300-mesh, nylon, 40 cm in diameter) method, with distilled water or physiological saline as the liquid to be absorbed. 0.10 g of hydrogel was put into the tea bag and then fully immersed in 1000 mL liquid at 30°C. After a predetermined time interval, the tea bag was taken out and dried in the air for 15 min. The hydrogel's swelling ratio was calculated using [26]

(1) $$\begin{array}{c} Q=\frac{W_{S}-W_{d}}{W_{d}}, \end{array}$$

where *W*_*s*_ and *W*_*d*_ are the weights of the swollen hydrogel and the dried sample, respectively. In this paper, *Q*_*d*_ (g·g^−1^) is the swelling in distilled water and *Q*_*s*_ (g·g^−1^) is the swelling ratio in physiological saline.

### 2.5. pH-Sensitive Properties of Hydrogels

Different pH buffer solutions with a pH range of 2–12 were used to study the pH sensitivity of hydrogels. NaOH and HCl were used to adjust the pH of buffer solution and the pH values were determined by a bench top pH-meter (METTLER TOLEDO, China). The dried hydrogel samples in tea bags were immersed in buffers for 24 h at 30°C, whilst (1) was used to calculate the equilibrium swelling ratio.

### 2.6. Temperature-Sensitive Properties of Hydrogels

The temperature-sensitive properties of the hydrogels were investigated by determining the swelling ratio in distilled water with different temperature. The samples were immersed in distilled water at a temperature ranging from 25 to 65°C. Subsequently, the hydrogels were removed and their weight was gravimetrically measured after eliminating the surface water. At each step, the water temperature was increased 5°C, and the hydrogels were kept in water for 30 min to ensure the equilibrium before the measurement of equilibrium swelling ratio [27].

### 2.7. Drug Loading and Release Behavior Studies

#### 2.7.1. Drug Loading

The drug loading property was evaluated by using salicylic acid as a drug model. To load drug into the hydrogels, 0.5 g of dry hydrogel was equilibrated in 100 mL salicylic acid solutions (with a concentration of 1 g/L) for 24 h at room temperature. The absorbance spectra of the supernatant solution were measured by a UV-VIS spectrophotometer (UV-1700 Hitachi High Technologies, Tokyo) at a wavelength 300 nm. The absorbance of salicylic acid with different concentrations was plotted as calibration curve for the calculation of drug's amount and the standard equation was calculated as *Y* = 0.0057 + 53.1*X* (*Y* is absorbance and *X* is concentration).

#### 2.7.2. Drug Release

To study the releasing behavior for the drug-loaded hydrogels, drug-loaded samples were immersed in buffer solutions of pH = 1.2 (simulated gastric fluid) and 7.4 (simulated intestinal fluid) at 37°C. At predetermined time intervals, 5 mL of the buffer solution was taken out for UV-VIS spectroscopy test and the concentration of the released drug was determined by recording the absorption spectra at 300 nm. Then the solution was replaced with fresh buffer solutions and the total volume maintains the same. The drug release efficiency was determined by

(2) $$\begin{array}{c} \text{Drug release}\left(\%\right)=\frac{C\left(t\right)}{C\left(0\right)}\times 100\%, \end{array}$$

where *C*(0) and *C*(*t*) are the amount of drug loaded and released at time *t*, respectively. All studies have been repeated in triplicate with 3 different samples.

#### 2.7.3. Kinetics Analysis of Drug Release

In order to understand the mechanism of salicylic acid release from the drug-loaded hydrogels, the power law equation which has been expressed by Korsmeyer–Peppas [28] can be described as

(3) $$\begin{array}{c} \frac{M_{t}}{M_{\infty }}={kt}^{n}, \end{array}$$

where *M*_*t*_ and *M*_*∞*_ were the amount of drug released at time *t* and at infinite time, respectively, *k* denotes the kinetic constant, and *n* values indicate the diffusion exponent of the mechanism of drug release [29]. Fickian diffusion dominates the release process when *n* < 0.45. The diffusion of model drug is from the hydrogel's access, which is formed during the swelling process in solution. Case II transport occurs at *n* > 0.89, where the relaxation or degradation of polymers happens. When 0.45 < *n* < 0.89, anomalous transport is observed which is driven by a coupling of Fickian diffusion and polymer relaxation/degradation, and the release is dominated by non-Fickian diffusion [30].

## 3. Results and Discussion

### 3.1. FTIR Analysis

**Figure 2:**
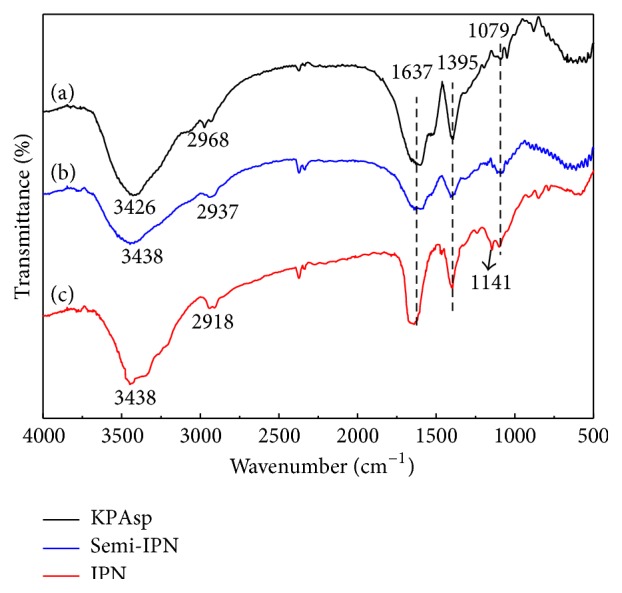
FTIR spectra of the hydrogels: KPAsp (a), KPAsp/PVA semi-IPN (b), and KPAsp/PVA IPN (c).

Figure 2 shows the FTIR spectra [31, 32] of KPAsp, KPAsp/PVA semi-IPN, and KPAsp/PVA IPN hydrogels, respectively. In curve (a), the strong broadband at 3426 cm^−1^ is due to O–H stretching, while the amidocyanogen (N–H) stretching also appears in the place. The absorption peak at 2937 cm^−1^ is due to –CH_2_– asymmetrical stretching vibration. The bands at 1637 and 1395 cm^−1^ are related to the symmetric and asymmetric stretching vibration of C=O, respectively. The peak at 1079 cm^−1^ can be ascribed to the symmetric stretching vibration of Si–O–Si that was formed through KPAsp self-cross-linking. These peaks can prove the presence of KPAsp. Compared with (a), the band at 3426 cm^−1^ shifted to 3438 cm^−1^ in curve (b), and this could be attributed to substantive hydroxyl groups due to the introduction of PVA. The appearance of 1141 cm^−1^ in curve (c) represented the stretching absorption peak of C–O–C, and this ether link was generated by the cross-linking reaction of glutaraldehyde with PVA. Moreover, carbonyl ester group was not observed at 1,735–1,750 cm^−1^, indicating that the synthesis process forms no new covalent bonds in the IPN hydrogels and the hydrogels' structure was formed through a physical process [24]. The above analysis indicated the semi-IPN and the IPN structure of KPAsp and PVA had been formed.

### 3.2. Morphology Analysis

**Figure 3:**
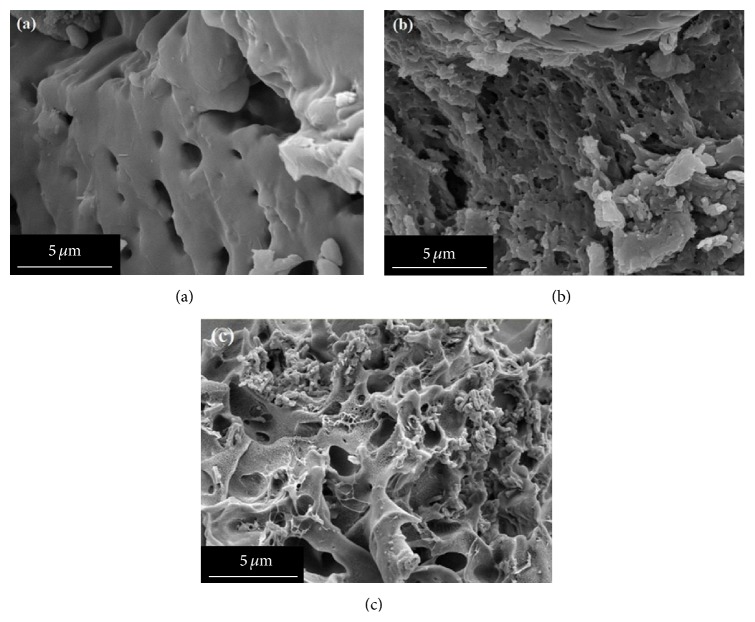
SEM images of KPAsp (a), KPAsp/PVA semi-IPN (b), and KPAsp/PVA IPN (c) hydrogels.

Figure 3 shows the SEM images of KPAsp, KPAsp/PVA semi-IPN, and KPAsp/PVA IPN hydrogels. It was found that the morphology of KPAsp hydrogel (Figure 3(a)) exhibited a smooth surface, while the surfaces of semi-IPN (Figure 3(b)) and IPN (Figure 3(c)) hydrogels were much rougher. The surface roughness of IPN hydrogel was the highest among the three samples. In addition, compared with KPAsp hydrogel, the semi-IPN and IPN hydrogels showed substantially enhanced pore densities. These phenomena indicate that the introduction of semi-IPN and IPN during hydrogel preparation can markedly improve the pore morphology and surface roughness of the KPAsp hydrogel. These pores as transport channels not only facilitate the diffusion of water and saline solution but also enlarged specific surface area in the hydrogel, which increased the contact probability of the hydrogel and water molecules. As the internal spaces of the hydrogels are essential for promoting effective swelling behavior [33], the IPN hydrogel with high pore density exhibits excellent swelling behavior. On the other hand, this IPN structure is helpful to increase in the equilibrium swelling ratio due to the formation of the second network that increased the effective network structure of hydrogels. This means the morphology of hydrogels could be improved by IPN process, which in turn provides a convenient way to improve the swelling properties of hydrogels.

### 3.3. Thermogravimetric Analysis

**Figure 4:**
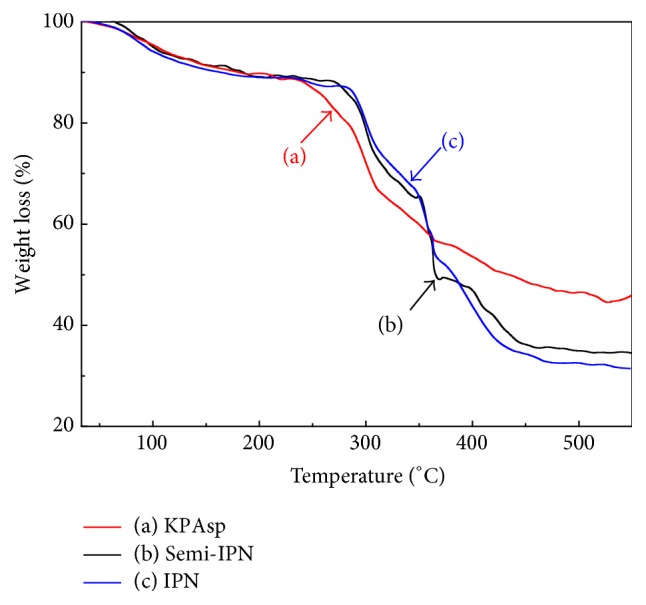
TGA patterns of KPAsp (a), KPAsp/PVA semi-IPN (b), and KPAsp/PVA IPN (c) hydrogels.

Thermal stability of KPAsp, KPAsp/PVA semi-IPN, and KPAsp/PVA IPN hydrogels was investigated by TGA. As shown in Figure 4, the three samples had a small quantity of weightlessness below 200°C, which was ascribed to the loss of surface moisture and bonded water. The thermal decomposition of semi-IPN hydrogels and IPN hydrogel initiated at 278°C and 286°C, whereas the KPAsp hydrogels were thermally decomposed at 244°C. Thus, the initial weight loss temperature of semi-IPN and IPN samples increased by 34°C and 42°C than that of KPAsp hydrogel, indicating that IPN structure significantly improved the thermal stabilities of hydrogels. These phenomena may attribute to the following: in the semi-IPN structure, the PVA chains were dispersed into the network of KPAsp. Since there is constraint of hydrogen bonds, the semi-IPN sample is not easy to decompose. Moreover, the PVA chains in the IPN sample form network structure through cross-linking of glutaraldehyde [34], which leads the thermal stabilities of IPN sample to become higher than that of the semi-IPN hydrogel.

### 3.4. Swelling Behavior Studies

**Figure 5:**
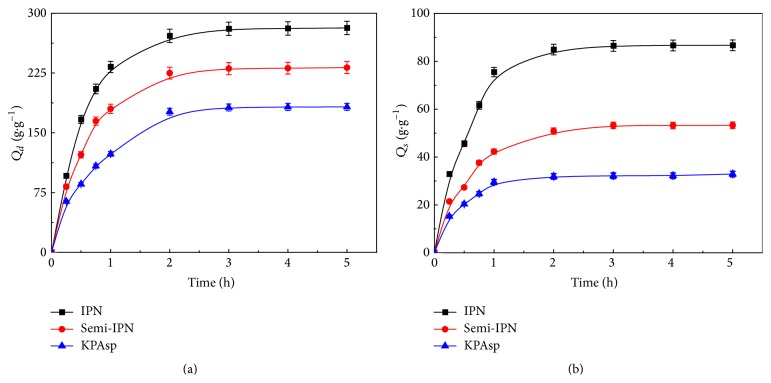
Swelling ratio of KPAsp, KPAsp/PVA semi-IPN, and KPAsp/PVA IPN hydrogels in distilled water (a) and 0.9 wt% physiological saline (b).

The dynamic swelling behavior of KPAsp, KPAsp/PVA semi-IPN, and KPAsp/PVA IPN hydrogels in distilled water and physiological saline was depicted in Figure 5. Each datum is an average of three independent measurements. The saturated swelling was obtained after 4 h. All the samples demonstrated similar swelling behavior. Initially, the swelling ratio of hydrogels abruptly rose and then began to level off. The IPN hydrogel had the maximal saturated swelling ratio both in distilled water and in physiological saline. The results clearly indicated semi-IPN or IPN structure was beneficial to the increase of swelling capacity. In the case of KPAsp/PVA semi-IPN hydrogel, PVA as large molecules with long chain impenetrated into the cross-linked network of KPAsp hydrogel, and the non-ion type hydrophilic group hydroxyl was introduced during this process. The common-ion effect and salt effect were weakened due to the introduction of the hydrophilic group –OH. As a result, the swelling ratio of KPAsp/PVA semi-IPN hydrogel is higher than that of KPAsp hydrogel. For KPAsp/PVA semi-IPN hydrogel, the water absorption of PVA is due to the hydration between water molecules and the hydrophilic –OH group, whereas the KPAsp/PVA IPN hydrogel and the large molecular chain of PVA were cross-linked with the ether bond and formed the second network structure. The water absorption of PVA is due to the hydration and the relaxation/expansion of the PVA network structure. Furthermore, glutaraldehyde only cross-links with PVA; thus the formation of the second network does not affect the network structure of the KPAsp. Contrary to the KPAsp/PVA semi-IPN hydrogel, there exists even more effective network structure in the IPN hydrogel, which results in its increase of swelling ratio both in distilled water and 0.9 wt% physiological saline. It is also clearly seen in Figure 5 that IPN hydrogel exhibits a faster swelling rate than semi-IPN and KPAsp hydrogel. The swelling rate of KPAsp hydrogel is about 124 g/g within 60 min, while the swelling rate of IPN hydrogel is about 233 g/g. This can be explained by the fact that the increased porous structure and surface area of the IPN hydrogel enhanced the diffusion of water into the hydrogel network [35]. Compared with KPAsp/PVA semi-IPN, the swelling rate of IPN hydrogel is higher. This is due to the presence of more effective network structure in IPN hydrogel which contains more water molecules.

### 3.5. Sensitivity of pH, Temperature, and Salt

#### 3.5.1. pH Sensitivity

**Figure 6:**
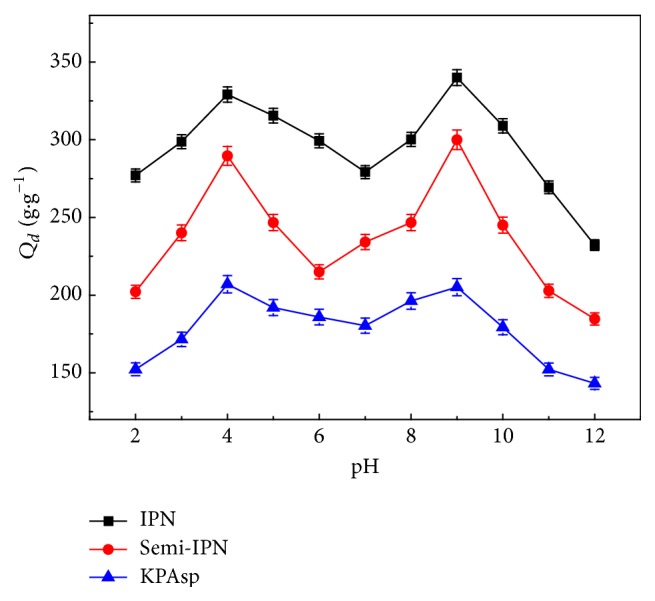
Swelling ratio of KPAsp, KPAsp/PVA semi-IPN, and KPAsp/PVA IPN hydrogels under different pH value.

The results of pH-dependent equilibrium swelling ratio of KPAsp, KPAsp/PVA semi-IPN, and KPAsp/PVA IPN hydrogels in the solution between 2 and 12 are shown in Figure 6. It can be seen that the swelling ratio reaches a maximum value when pH = 4 and 9. KPAsp hydrogel is derived from L-aspartic acid, which is partially acidic ampholyte due to the presence of amino acids. Two sharp water absorption peaks were observed which are attributed to the strong repulsion of –NH_2_^+^ groups in acidic medium and –COO^−^ groups in basic solution, respectively. At acidic condition (pH < 4), one can notice an obviously reduced swelling; it was aroused by the screening effect of the counter ions (e.g., Cl^−^) which reduce the effective charges of –NH_2_^+^ and therefore decrease the efficient repulsion between the two cations (–NH_2_^+^) [36]. However, in a pH near neutral (e.g., pH = 6–8), most acid and base groups are nonionized. Therefore, the hydrogen bonding between amine and carboxylic acid/carboxamide groups forms cross-linking and leads to a decreased swelling ration of the hydrogel. When pH increases, a high swelling ratio was observed due to the increasing electrostatic repulsive forces between the deprotonated –COO^−^ groups. When pH > 9, the screening effect of Na^+^ hinders the swelling of hydrogels. To sum up, the equilibrium swelling ratio of pH-sensitive hydrogels is influenced by the hydrogen bonds and attractive/repulsive electrostatic interactions among the functional groups in different pH conditions.

On the other hand, according to the Donnan equilibrium theory [37, 38], the swelling of hydrogel is mainly determined by a balance of osmotic pressure between the internal and external medium of the hydrogel network. Amine (carboxamide) and carboxylate groups are the main functional groups in KPAsp hydrogel. At pH < 4, secondary amine groups and carboxylic groups are protonated to –NH_2_^+^ and –COOH, respectively, whilst at pH > 9, –COOH deprotonates to –COO^−^. Due to the presence of –NH_2_^+^ or –COO^−^ groups in hydrogels, the charge density on the polymer increases and a higher osmotic pressure occurs in the hydrogel aroused from the repulsion between the cations (–NH_2_^+^) or anions (–COO^−^) [36]. The balance of osmotic pressure also leads to the swelling of KPAsp hydrogel. Furthermore, the introduction of non-ion type hydrophilic group –OH and IPN structure does not change the swelling trend of KPAsp hydrogel at various pH and only improves the swelling ratio of the IPN hydrogel.

#### 3.5.2. Temperature Sensitivity

**Figure 7:**
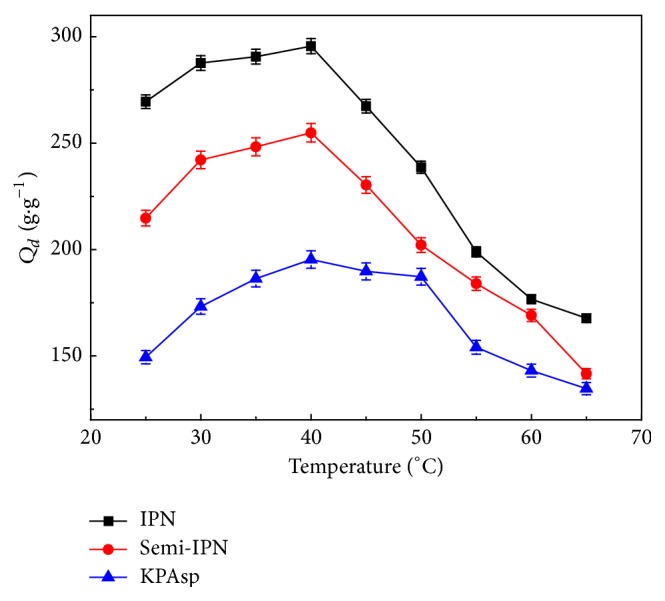
Swelling ratio of KPAsp, KPAsp/PVA semi-IPN, and KPAsp/PVA IPN hydrogels with the variation of temperature.

Temperature is an important environmental factor to appraise a hydrogel.  Figure 7 depicts the temperature-dependent swelling of KPAsp, KPAsp/PVA semi-IPN, and KPAsp/PVA IPN hydrogels in a temperature range of 25 to 65°C. It can be seen that the swelling ratio was firstly increased with the increasing temperature and then fell down quickly, indicating that all hydrogels were thermosensitive. The temperature of optimum swelling ratio for all samples was 40°C. The swelling ratio of semi-IPN and IPN hydrogels was always higher than that of KPAsp hydrogel.

This phenomenon can be ascribed to the interaction of molecular bandings with the network structure [39]. As mentioned previously, strong hydrogen bonding was produced among the carboxyl, hydroxyl, and imide groups in the polymer molecular chains. The introduction of PVA into hydrogel can efficiently enhance the number of hydroxyls which strengthens intermolecular forces. If the hydrogel was put in a low temperature, these H-bonds compress the network structure to a shrinking and bending status, which results in a minor swelling ratio. As the temperature increases, the hydrogen bond effects were weakened and the twisted polymer molecular chains extend gradually. As a consequence, the probability of polymer molecular chains contacting with water molecule grew larger; thus the swelling ratio increased slowly. However, when the temperature was too high, the network structure collapses due to the hydrogen bonds breaking between the water molecules and PVA in the network, and the swelling ratio decreases rapidly. The capability of the thermosensitivity of IPN hydrogels was enhanced, indicating there was alteration in the structure of hydrogels through semi-IPN and IPN technique.

#### 3.5.3. Salt Sensitivity

**Figure 8:**
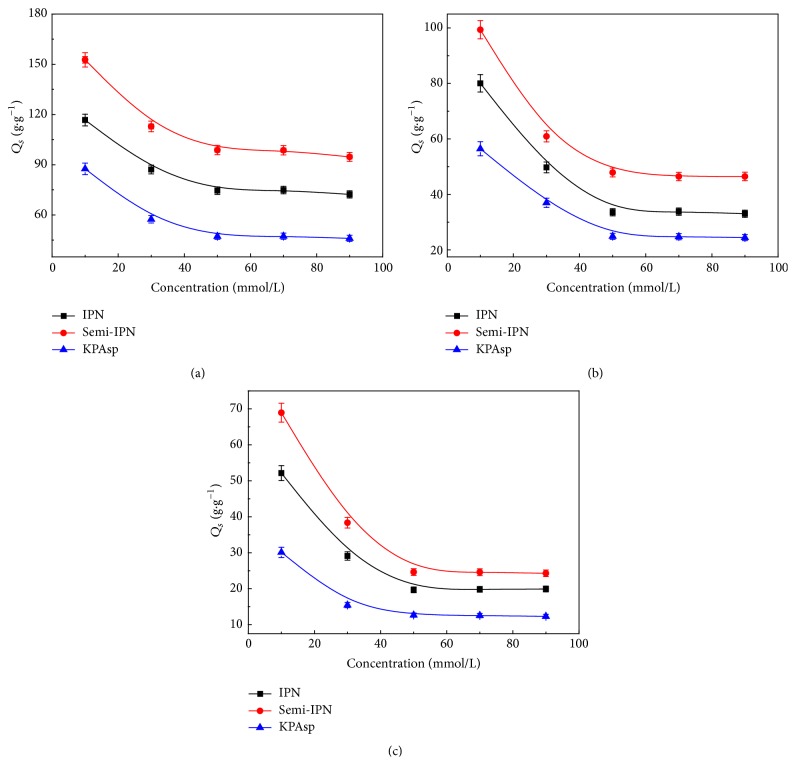
Swelling ratio of KPAsp, KPAsp/PVA semi-IPN, and KPAsp/PVA IPN hydrogels in different salt solutions: NaCl (a), CaCl_2_ (b), and FeCl_3_ (c).

The effect of salt solutions on swelling ratio of three hydrogels with different structure was shown in Figures 8(a), 8(b), and 8(c), corresponding to the result of NaCl, CaCl_2_, and FeCl_3_ solution, respectively. It was observed that the swelling ratios of all hydrogels decreased with the increase of both salt concentration and the charge of the cation.

In the same salt solution, the ionic strength of solution increased with the concentration, while, for the three chloride salt solutions at the same concentration (NaCl, CaCl_2_, and FeCl_3_), the ionic strength lists in the order of cation charge Na^+^ < Ca^2+^ < Fe^3+^. The relationship between the swilling ratio and the ionic strength in 10 mmol/L salt solution is included in Table 1.

The effect of the ionic strength of the external solution on the swelling behavior has been described by Hermans as follows [40]:

(4) $$\begin{array}{c} Q^{5/3}\approx \frac{{\left(i/2V_{u}S^{1/2}\right)}^{2}+\left(1/2-x_{1}\right)/V_{1}}{{V_{e}/V}_{0}}, \end{array}$$

where *Q* is the swelling ratio, *V*_*e*_/*V*_0_ is the effectively cross-linked chains in the unit volume, *S* is the ionic strength of the swollen liquid, *i*/*V*_*u*_ is the concentration of the fixed charge of the unswollen networks, *x*_1_ is the polymer-solvent thermodynamic interaction parameter, and *V*_1_ is the molar volume of water. According to this equation, the water absorbency of the hydrogels decreases with the ionic strength of external solution. In the swelling process, the anions on the hydrogels ionize initially and the hydrogel surface swells due to the electrostatic repulsion of the ionized groups. When ionic strength in the solution increases, the anionic groups are screened by the cations in the solution, and the swelling ratio decreases. Another possible explanation is that Cl^−^ ions in the solution swamp the negatively charged –COO^−^ groups.

The complexation between the multivalent cations and the –COOH groups in KPAsp generated ionic cross-linking in the hydrogels. With ionic strength increasing, the ionic cross-linking increased. As a result, the conformation of the hydrogel was changed from an expanded structure to a more compact matrix, which induced the swelling ratio decrease. According to Flory's theory [41], the swelling ratio decreases with the further augment of the cross-linking density in the hydrogels.

In the same ionic strength for a given salt solution, the swelling ratio of IPN hydrogel has the highest value, which demonstrates that the IPN structure could improve the salt tolerance of hydrogels. There were two reasons for the high swelling ratio of IPN hydrogel. The complexation between multivalent cations and –COOH groups is weakened due to the introduction of the hydrophilic group –OH, and PVA cross-linking forms the second network structure, which increased the effective network structure of hydrogels.

### 3.6. Drug Loading and Release Behavior Studies

The amounts of salicylic acid loaded in KPAsp, KPAsp/PVA semi-IPN, and KPAsp/PVA IPN hydrogels were measured to be 36.69 mg/g, 48.05 mg/g, and 65.67 mg/g, respectively. Similar to the swelling behavior of KPAsp, KPAsp/PVA semi-IPN, and KPAsp/PVA IPN hydrogels in distilled water, the higher salicylic acid loaded in the KPAsp/PVA IPN hydrogel attributed to the introduction of hydrophilic group –OH and the increase of effective network structure of the IPN hydrogel. Moreover, the increased porous structure could also enhance the diffusion of drug into the hydrogel network.

**Figure 9:**
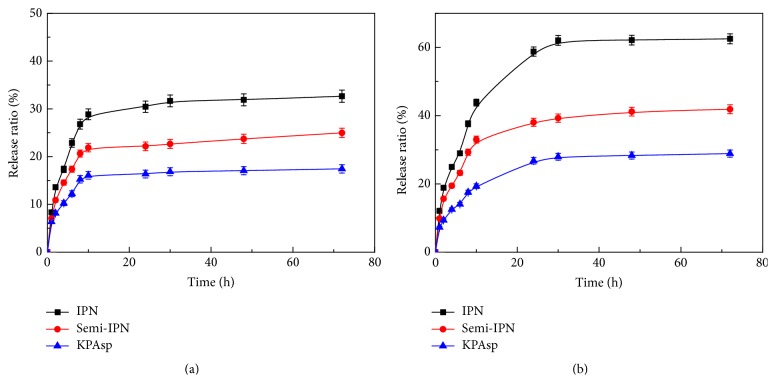
Release behavior of KPAsp, KPAsp/PVA semi-IPN, and KPAsp/PVA IPN hydrogels in pH values at 1.2 (a) and 7.4 (b).

Figure 9 presented the release profiles of salicylic acid from the three hydrogels at pH values of 1.2 (simulated gastric fluid) and 7.4 (simulated intestinal fluid) at 37°C. As clearly shown in Figure 9, all the hydrogels can be observed with significant pH-dependent response. The drug release of each hydrogel in pH values of 1.2 was obviously slower than that in pH values of 7.4. In addition, the release rate of salicylic acid from the KPAsp/PVA IPN hydrogel is always the highest among the three hydrogels, which should be related to the IPN structure with increased porous structure and specific surface area [42]. The corresponding cumulative amounts of salicylic acid released from the KPAsp/PVA IPN hydrogel were 32.6% at pH 1.2, while the cumulative release rate reached 62.5% at pH 7.4. That may be ascribed to the increase of –OH on the hydrogel with the PVA introduction. In acid environment carboxyl groups on the IPN hydrogel are in the form of –COOH, and the effect of H-bonding between these –COOH and –OH limits the release of salicylic acid. As the pH value increases, carboxyl groups on the IPN hydrogel incline to form –COONa, and the effect of H-bonding between –COOH and –OH recedes; therefore salicylic acid becomes easy release from the hydrogel. In conclusion, this novel IPN hydrogel can control drug release by external surroundings stimuli and has a relative high drug release rate in simulated intestinal fluid.

**Figure 10:**
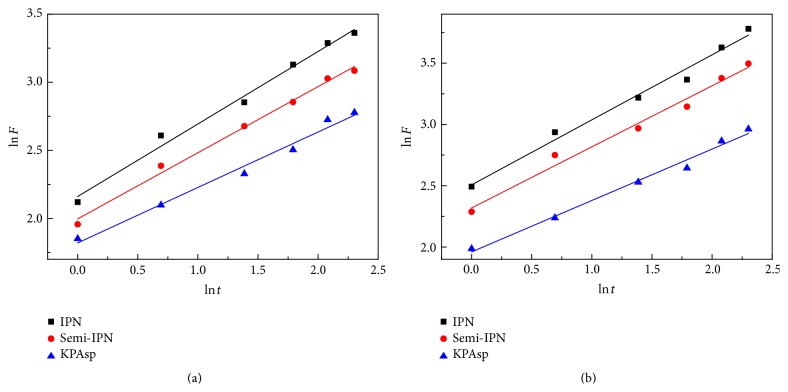
Release kinetics (ln⁡*t*-ln⁡*F* fitting curve) of KPAsp, KPAsp/PVA semi-IPN, and KPAsp/PVA IPN hydrogels in pH values at 1.2 (a) and 7.4 (b).

The salicylic acid release from the hydrogels in the first 10 h was further analyzed using Korsmeyer–Peppas equation [28]. The release kinetics (ln⁡*t*-ln⁡*F* fitting curve) of KPAsp, KPAsp/PVA semi-IPN, and KPAsp/PVA IPN hydrogels in pH values at 1.2 and 7.4 was indicated in Figure 10. And the calculated values of the diffusion exponent *n* and the kinetic constant *k* for all of the hydrogels and the correlation coefficients (*R* ^2^) of these data were shown in Table 2. These values distinctly demonstrated that the diffusion exponent *n* increased with the introduction of IPN. The mechanism of drug controlled release from KPAsp hydrogel followed a Fickian diffusion which indicated that diffusion of salicylic acid plays a major role rather than polymer relaxation or degradation. The salicylic acid release from the semi-IPN and IPN hydrogels followed a non-Fickian diffusion controlled mechanism, suggesting that the drug release was dominated by mechanism of Fickian diffusion and polymer relaxation. These results clearly indicated that IPN structure had an effect on the drug transport mechanism. This would be useful in the design and development of novel controlled delivery systems.

## 4. Conclusions

Novel biodegradable KPAsp/PVA IPN hydrogel was successfully synthesized in an aqueous system by a simple one-step method. During the synthesis process of IPN hydrogels, the self-cross-linking reaction of KPSI and PVA occurs simultaneously. Secondly, homogeneous network structure of IPN hydrogel was obtained in uniform reaction system. Thirdly, the properties of KPAsp and PVA components were integrated with IPN technology. Thus, the IPN hydrogels exhibit outstanding performance of swelling, drug release, and pH, temperature, and salt sensitivities compared to pure KPAsp hydrogels. SEM results confirmed that there appear porous structure and rough surface in IPN hydrogel. The swelling ratio of the IPN hydrogel in physiological saline and distilled water reached 86.7 g·g^−1^ and 281.6 g·g^−1^, which showed 1.64 times and 54.2% enhancement in comparison with that of KPAsp hydrogel. The KPAsp/PVA IPN hydrogel demonstrated ampholytic pH sensitivity and prominent sensitivity of temperature, ionic strength, and cationic kind. The study of drug-loaded characteristics of the hydrogels revealed that the IPN hydrogel showed the best drug-loaded capacity. The controlled drug release study demonstrated that drug release rate was controlled by the structure and pH sensitivity. Results of the release of salicylic acid from the three hydrogels indicated the drug release property of KPAsp/PVA IPN hydrogel is the most remarkable, and a relative large amount of drug released was preferred under intestinal fluid environment. This study will be useful for designing and developing novel controlled delivery systems.

## Figures and Tables

**Table 1:** Relationship between the ionic strength and swelling ratio.

Solution	Ionic strength (mmol/kg)	Swelling ratio (g·g^−1^)	
		IPN	Semi-IPN KPAsp/PVA
10 mmol/L NaCl solution	10	152.66	116.73
10 mmol/L CaCl_2_ solution	30	99.35	80.03
10 mmol/L FeCl_3_ solution	60	68.94	52.14

**Table 2:** Release kinetic parameters *n*, *k*, and *R* ^2^ for ln⁡*t*-ln⁡*F*.

Diffusion medium	Sample	*n*	*k*	*R* ^2^
pH = 1.2	KPAsp	0.4073	1.82	0.9807
	Semi-IPN	0.4858	1.9965	0.9919
	IPN	0.5326	2.1609	0.9873

pH = 7.4	KPAsp	0.4196	1.9599	0.9819
	Semi-IPN	0.4984	2.3187	0.9799
	IPN	0.5306	2.507	0.9819
